# SLC26A Gene Family Participate in pH Regulation during Enamel Maturation

**DOI:** 10.1371/journal.pone.0144703

**Published:** 2015-12-15

**Authors:** Kaifeng Yin, Yuejuan Lei, Xin Wen, Rodrigo S. Lacruz, Manoocher Soleimani, Ira Kurtz, Malcolm L. Snead, Shane N. White, Michael L. Paine

**Affiliations:** 1 Center for Craniofacial Molecular Biology, Herman Ostrow School of Dentistry of University of Southern California, Los Angeles, California, United States of America; 2 Department of Operative and Endodontics, The Affiliated Stomatological Hospital, Guangxi Medical University, Nanning, Guangxi, China; 3 Basic Science and Craniofacial Biology, New York University College of Dentistry, New York, New York, United States of America; 4 Department of Medicine, University of Cincinnati, Research Services, Veterans Affairs Medical Center, Cincinnati, Ohio, United States of America; 5 Division of Nephrology, David Geffen School of Medicine at University of California Los Angeles, Los Angeles, California, United States of America; 6 School of Dentistry, University of California Los Angeles, Los Angeles, California, United States of America; Department of Biomaterials, JAPAN

## Abstract

The bicarbonate transport activities of Slc26a1, Slc26a6 and Slc26a7 are essential to physiological processes in multiple organs. Although mutations of Slc26a1, Slc26a6 and Slc26a7 have not been linked to any human diseases, disruption of Slc26a1, Slc26a6 or Slc26a7 expression in animals causes severe dysregulation of acid-base balance and disorder of anion homeostasis. Amelogenesis, especially the enamel formation during maturation stage, requires complex pH regulation mechanisms based on ion transport. The disruption of stage-specific ion transporters frequently results in enamel pathosis in animals. Here we present evidence that Slc26a1, Slc26a6 and Slc26a7 are highly expressed in rodent incisor ameloblasts during maturation-stage tooth development. In maturation-stage ameloblasts, Slc26a1, Slc26a6 and Slc26a7 show a similar cellular distribution as the cystic fibrosis transmembrane conductance regulator (Cftr) to the apical region of cytoplasmic membrane, and the distribution of Slc26a7 is also seen in the cytoplasmic/subapical region, presumably on the lysosomal membrane. We have also examined *Slc26a1* and *Slc26a7* null mice, and although no overt abnormal enamel phenotypes were observed in *Slc26a1* ^*-/-*^ or *Slc26a7* ^*-/-*^ animals, absence of Slc26a1 or Slc26a7 results in up-regulation of *Cftr*, *Ca2*, *Slc4a4*, *Slc4a9* and *Slc26a9*, all of which are involved in pH homeostasis, indicating that this might be a compensatory mechanism used by ameloblasts cells in the absence of Slc26 genes. Together, our data show that Slc26a1, Slc26a6 and Slc26a7 are novel participants in the extracellular transport of bicarbonate during enamel maturation, and that their functional roles may be achieved by forming interaction units with Cftr.

## Introduction

Enamel development involves two major functional stages, secretory and maturation [1]. In the secretory stage, ameloblasts synthesize and secrete a number of structural Enamel Matrix Proteins (EMPs) [2–4]. The enamel matrix during the secretory stage is maintained at near-neutral pH conditions in a protein-rich environment. During the maturation stage, the extracellular pH varies considerably, ranging from neutral to acidic conditions, with a return to a more physiologic pH level at the end of maturation stage [4,5]. Changes in extracellular pH values require sophisticated regulatory mechanisms by ameloblasts, so as to maintain the acid-base balance in the microenvironment surrounding the apical pole to sustain crystal nucleation and growth [6]. In addition, extracellular EMPs at the maturation-stage are internalized by ameloblasts and degraded through endosome-lysosome pathways [7,8]. Regulation of intracellular pH is also required during endocytosis to create an acidic luminal environment for hydrolytic enzyme activation [9]. Although there is still uncertainty about the exact temporal and spatial working model of pH regulation during amelogenesis [2,3,6,10,11], the involvement of carbonic anhydrases (CAs), cystic fibrosis conductance transmembrane conductance regulator (CFTR), Chloride Channels (CLCNs), Solute Carrier family 4 (SLC4s) and Solute Carrier family 9 (SLC9s) in ameloblast-mediated pH homeostasis has been widely accepted [2,7,12–32].

The SLC26 gene family encodes multifunctional anion exchangers and anion channels with a broad range of substrates [33]. In mammals, this family consists of 11 genes, *SLC26A1-SLC26A11*. Based on our previous genome-wide miRNA and mRNA expression profiling of the enamel organ cells in rats [34], *Slc26a1*, *Slc26a6* and *Slc26a7* are the only members among the *Slc26a* gene family whose transcripts are significantly up-regulated during maturation-stage enamel formation when compared to secretory-stage [34]. *Slc26a1*, *Slc26a6* and *Slc26a7*, which code for the proteins Sat1, Pat1 and Sut2 respectively, all exhibit chloride/bicarbonate exchanger activities [35–38]. Mutations in *Slc26al*, *Slc26a6* or *Slc26a7* lead to multiple disorders, such as urolithiasis, hepatotoxicity, distal renal tubular acidosis and impaired gastric secretion, induced by the disruption of ion homeostasis [39–42]. Enamel maturation involves pH regulation mediated by multiple ion transport/exchange activities across plasma and endosome membranes [2,3,7,8,12–32,43–47]. Thus there is a need to better understand the functional activities of the SLC26A gene family members in amelogenesis.

In the present study, we conducted quantitative real-time PCR and Western blot analyses, and showed that Slc26a1, Slc26a6 and Slc26a7 are all significantly up-regulated at maturation stage compared with secretory stage at both the mRNA and protein levels. Based on immuolocalization data, we show that in maturation-stage ameloblasts, the gene products of *Slc26a1*, *Slc26a6* and *Slc26a7* localize to the apical region of the cytoplasmic membrane, similar to the localization pattern of Cftr in maturation-stage ameloblasts. In addition, Slc26a7 is also seen within the cytoplasmic/subapical region of ameloblasts, presumably on the lysosomal membrane. From the protein complex pulled down using an antibody to Cftr, we detected Slc26a1, Slc26a6 and Slc26a7 via immunoblotting, suggesting the direct interaction of each of these three Slc26 proteins with Cftr. Compared with wild-type (WT) animals, *Slc26a1* ^*-/-*^ and *Slc26a7* ^*-/-*^ animals did not show any clearly noticeable abnormalities in the mature enamel phenotype (density and structure). However, many gene transcripts examined by real-time PCR–such as Car2 (carbonic anhydrase 2), Cftr, Slc4a4/NBCe1, Slc4a9/Ae4, Slc26a9 and Alpl (alkaline phosphatase)–showed significant up-regulation in the enamel organ cells of *Slc26a1* ^*-/-*^ and *Slc26a7* ^*-/-*^ animals when compared to age- and sex-matched wild-type controls. Collectively, these data indicate that Slc26a1, Slc26a6 and Slc26a7 are actively involved in ion transport related to pH regulation processes during enamel maturation and their functional roles may be achieved, at-least in part, by forming protein supramolecular assemblies by their interactions with Cftr.

As mentioned above, for the ion channels discussed here the names assigned to the genes are different from the names assigned to their products. For example *Slc4a4*, *Slc26a1*, *Slc26a6* and *Slc26a7* code for proteins AE2, Sat1, Pat1 and Sut2 respectively. To avoid confusion, in this paper we will refer to both the genes and their respective gene products (mRNA and protein) by their official gene ID rather than the product name.

## Materials and Methods

### Animals

**Fig 1:**
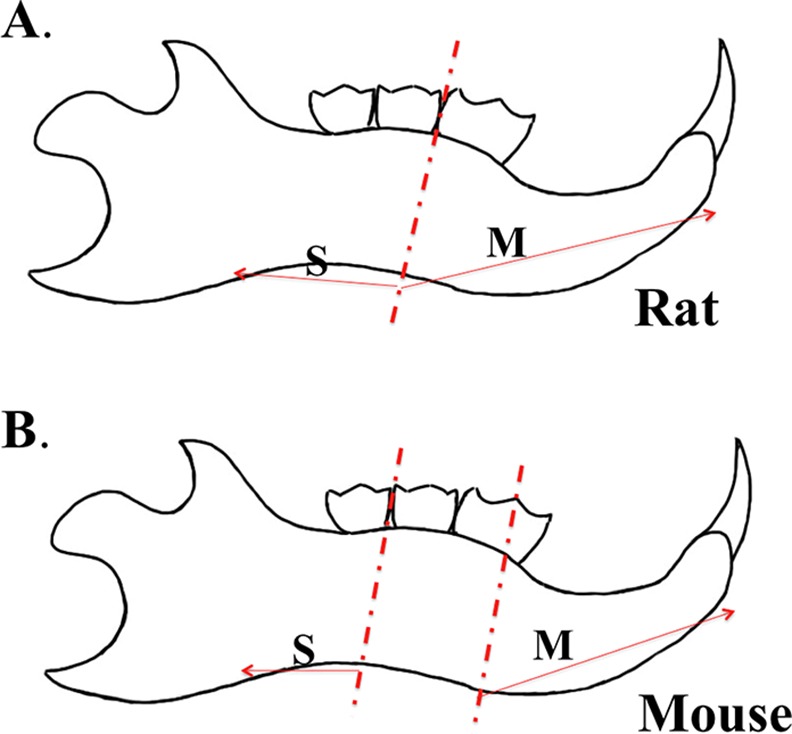
Reference lines for animal dissection. A. In 4-week-old rats, the secretory- (S) and maturation-stage (M) enamel organs along the enamel surface of mandibular incisor are separated by the reference line between the first and the second molar [3,43]. B. Two reference lines were used to partition secretory-stage (S) from maturation-stage (M) enamel organ. The first reference line, vertical to the inferior border of mandibular cortical bone, divided the mesial-distal width of the first molar into halves. The second reference line, also vertical to the inferior to the bony border of the mandible, was located between the second and the third molar.

All vertebrate animal studies complied with Institutional and Federal guidelines. For real-time PCR, western blot and co-immunoprecipitation analyses, we obtained RNA and protein samples from the enamel organs lining the surface of rat (Wistar Hannover, 4-week, 100–110g) incisors, because the reference line separating the secretory- and the maturation-stage enamel organs has been well documented in rats [3,43] (Fig 1). The subsequent immunohistochemistry and immunofluorescence detection of target gene products were also conducted on sections of rat mandibles. *Slc26a1* ^*+/-*^ mice were purchased from the Jackson Laboratory (stock # 012892) [39]. *Slc26a1* ^*-/-*^ mice were generated by breeding heterozygous (*Slc26a1* ^*+/-*^) parents. *Slc26a7* ^*+/-*^ mice were a kind gift from Dr. Manoocher Soleimani [42], and bred in an identical manner to the *Slc26a1* mutants. Although *Slc26a7* null animals have been documented to show distal renal tubular acidosis and impaired gastric acid secretion, they were still reported to exhibited normal growth and survival compared to their WT littermates [42]. Both *Slc26a1* ^*-/-*^ and *Slc26a7* ^*-/-*^ mice are viable, fertile and normal in physical size compared with their WT and heterozygous littermates. *Slc26a1* ^*-/-*^ and *Slc26a7* ^*-/-*^ animals were genotyped by PCR using primers designed in earlier studies [39,42].

### Rat tissue dissection, RNA extraction & real-time PCR analysis

For semi-quantifying the expression of Slc26a1, Slc26a6 and Slc26a7 mRNA, secretory-stage and maturation-stage RNA samples were obtained from the enamel organs of rat incisors. Four 4-week old Wistar Hannover rats, each weighing 100–110g, were sacrificed for their mandibles. The rat mandibles were kept in liquid nitrogen overnight and then lyophilized for over 24 hours. After removing the cortical bone enclosing the incisors, the exposed multi-cellular layers along the enamel surface were collected into RNase-free Eppendorf tubes. Details regarding the dissection procedures were described previously [43,44]. The total RNA was extracted separately from secretory-stage and maturation-stage enamel organs using the miRNeasy Mini Kit (Qiagen, Valencia, CA, USA). The RNA sample from each of the four rats was also processed separately. cDNA used for real-time PCR analysis was synthesized using the miScript II RT Kit with miScript HiFlex Buffer (Qiagen). In order to check the accuracy of dissection, the expression of two stage-specific genes, *Odam* (highly expressed during maturation stage) and *Enam* (highly expressed during secretory stage), were each subjected to real-time PCR analysis [24,43] before proceeding to examining *Slc26a1*, *Slc26a6* and *Slc26a7*, and other related gene transcript profiles. Real-time PCR reactions were performed on the CFX96 Touch^TM^ Real-Time PCR Detection System (Bio-rad Life Sciences, Hercules, CA) with iQ SYBR® Green supermix (Bio-rad Life Science) and rat-specific primers (S1 Table). The raw data acquired were in the form of Ct values, which were normalized to the Ct values of Actb (β-actin). The ΔΔCt method was used to calculate the fold changes in the expression of *Slc26a1*, *Slc26a6* and *Slc26a7* (maturation stage relative to secretory stage) [48,49]. Two-tailed Student’s t tests were used to examine potential differences in the expression levels of Slc26a1, Slc26a6 and Slc26a7 transcripts between secretory- and maturation-stage (α = 0.05). Data were analyzed using IBM SPSS Statistics 22.0 software (IBM Corporation, Armonk, NY, USA).

### Mouse tissue dissection, cDNA analysis and real-time PCR

The mandibles were isolated from 4-week *Slc26a1* ^*+/+*^ and *Slc26a1* ^*-/-*^, and *Slc26a7* ^*+/+*^ and *Slc26a7* ^*-/-*^ mice. The mandibles were then processed separately using the same procedures as those used for rat mandibles [43,44]. For RNA extraction from mouse mandibles, we used different reference lines from those used for the extraction from rat mandibles (Fig 1). On the lingual side of the mouse hemi-mandible, the first reference line, vertical to the inferior border of mandibular cortical bone, cut approximately half of the mesial-distal width of the first molar. The second reference line, also vertical to the inferior to the bony border of the mandible, was located between the second and the third molar (Fig 1). We collected the multi-cellular layers on the surface of incisor enamel mesially of the first reference line as maturation-stage-derived enamel organ and the tissues distally of the second reference line as secretory-stage-derived enamel organ (Fig 1). The dissected RNA samples from secretory and maturation stages were validated by detecting the stage-specific expression of *Odam* and *Enam* (relative to *Actb*) using real-time PCR. cDNA used for real-time PCR analysis was prepared using the miScript II RT Kit with miScript HiFlex Buffer (Qiagen). Real-time PCR reactions were performed on the CFX96 Touch^TM^ Real-Time PCR Detection System (Bio-rad Life Sciences) with iQ SYBR® Green supermix (Bio-rad Life Science) and mouse-specific primers (S1 Table). In order to verify that there were no intact transcripts of *Slc26a1* or *Slc26a7* in the maturation-stage enamel organs of mutant animals, PCR reactions were conducted using the maturation-stage cDNA template and self-designed primers. The sequences of the primers were: Slc26a1 Forward 5`-cctggatattgcaaagccttcag-3`, Slc26a1 Reverse 5`-gaatcctgggaagggtcaaagc-3`(product 524 bp); Slc26a7 Forward: 5`-cgggagcaaagaggaaaaag-3`, Slc26a7 Reverse: 5`-gtaagcaggaatgtggcactg-3`(product 520 bp). The PCR reactions were set as follows: for Slc26a1, 94°C initial denaturation (10 min), 35 cycles at 95°C (1 min), 59°C (1 min), 72°C (1 min), 72°C (8 min, final extension), followed by a return to 4°C; for Slc26a7, 94°C initial denaturation (10 min), 35 cycles at 95°C (1 min), 58°C (1 min), 72°C (1 min), 72°C (8 min, final extension), followed by a return to 4°C. No PCR products with expected sizes were generated in mutant animals. In addition, real-time PCR reactions were performed to detect the expression changes of the genes that are or might be involved in maturation-stage regulation (S1 and S2 Tables) [7,8,12–32,43–53], using mouse-specific primers (S1 Table) and cDNA samples from *Slc26a1* ^*+/+*^, *Slc26a1* ^*-/-*^, Slc26a7^+/+^ and Slc26a7^-/-^ animals separately. The procedures of data analysis for real-time PCR were as described above for rat tissue analysis. The relative expression levels of each gene were evaluated between the *Slc26a1* ^*+/+*^ and *Slc26a1* ^*-/-*^ animals and between the Slc26a7^+/+^ and Slc26a7^-/-^ animals using two-tailed Student’s t tests in IBM SPSS Statistics 22.0 (α = 0.05).

### Immunoperoxidase immunohistochemistry (IHC)

Wistar Hannover rats (100–110g body weight, 4 weeks old) were sacrificed for their mandibles. The hemi-mandibles were then fixed in 4% paraformaldehyde (PFA) at 4°C overnight. 10% EDTA (pH 7.4) was used to decalcify the samples for 10–12 weeks. 8μm sagittal sections were prepared from paraffin-embedded samples. After the tissue sections were dewaxed and rehydrated, endogenous peroxidase was blocked by 0.3% H_2_O_2_ in methanol. Sections were blocked by 1% bovine serum albumin (BSA) in PBS (1X, pH 7.4) and incubated overnight with the primary antibodies against Slc26a1, Slc26a6 or Slc26a7 (antibody sources are listed in Table 1). Tissue sections were counter-stained using Mayer`s hematoxylin after an 3-Amino-9-ethylcarbazole (AEC)/ 3, 3'-diaminobenzidine (DAB) staining kit was applied (Table 1). Negative controls were sagittal sections subjected to all staining procedures but with no antibodies added.

**Table 1:** Antibodies used for western blot, immunoperoxidase immunostaining, immunofluorescence and co-immunoprecipitation analyses.

Manufacturer (catalog #)	Gene product detected	Application
Proteintech (10708-1-AP)	Slc26a1 (rat)	immunohistochemistry (dilution 1:300)
Santa Cruz Biotechnology (sc-132090)	Slc26a1 (rat)	immunofluorescence (dilution 1:400)
		western blot/co-immunoprecipitation (dilution 1:100)
Abcam (ab99559)	Slc26a6 (rat)	Immunohistochemistry (dilution 1:5000)
Santa Cruz Biotechnology (sc-26728)	Slc26a6 (rat)	Immunofluorescence (dilution 1:100)
		western blot/co-immunoprecipitation (dilution 1:100)
Santa Cruz Biotechnology (sc-53960)	Slc26a7 (rat)	immunohistochemistry (dilution 1:100)
Abcam (ab65367)	Slc26a7 (rat)	immunofluorescence (dilution 1:300)
		western blot/co-immunoprecipitation (dilution 1:1000)
Abcam (ab42687)	Ae2 (rat)	immunofluorescence (dilution 1:100)
Abcam (ab24170)	Lamp1 (rat)	immunofluorescence (dilution 1:1000)
Santa Cruz Biotechnology (sc-8909)	Cftr (rat)	immunofluorescence (dilution 1:100)
		co-immunoprecipitation (amount 1μg)
Abcam (ab6276)	Actb (rat)	western blot (dilution 1:3000)

### Co-localization analysis by Immunofluorescence (IF)

With the purpose of clarifying the localization of Slc26a1, Slc26a6 and Slc26a7 within the milieu of maturation-stage ameloblasts, we conducted immunofluorescence (IF) to co-localize Slc26a1, Slc26a6 and Slc26a7 with other gene products–Ae2, Lamp1, and Cftr–whose localization in ameloblasts have been previously reported [7,8,13–15,18,24,26,46,53]. The protocols of preparing tissue sections for IF were the same as those used for immunoperoxidase IHC. BSA-blocked tissue sections were incubated overnight with different combinations of primary antibodies: Slc26a1 x Ae2; Slc26a1 x Lamp1; Slc26a6 x Ae2; Slc26a6 x Lamp1; Slc26a7 x Ae2; Slc26a7 x Lamp1; and Slc26a7 x Cftr (Table 1). The co-localization analysis was not conducted for the combinations of Slc26a1 x Cftr, or Slc26a6 x Cftr, as the antibodies to Slc26a1, Slc26a6 and Cftr were all goat-derived and therefore not suitable (Table 1), and no commercially available non-goat-derived Cftr antibodies suitable for rodent tissues could be identified. All tissue sections used for co-localization analyses were stained with DAPI (Vector Laboratories; Catalog # H-1200) before the cover slips were added.

### Western blot analysis

Protein samples were obtained from three Wistar Hannover rats (100–110g body weight, 4 weeks old, n = 3). After the animals were euthanized and decapitated, the mandibles were dissected and isolated immediately. We used the reference line between the first and the second molar to discriminate secretory-stage from maturation-stage enamel organ [3,43] (Fig 1). The bony structures enclosing the incisor were removed and the enamel organs along the enamel surface were collected to analyze secretory stage and maturation stage separately. The samples were added to pre-cooled Eppendorf tubes containing RIPA Lysis and Extraction buffer (Thermo Fisher Scientific Inc., Rockford, IL, USA; Catalog # 89901) mixed with Halt Protease Inhibitors Cocktail (Thermo Fisher Scientific; Catalog # 78429). The samples were homogenized with a pestle (on ice), kept on ice for 30min and centrifuged at 16,000rpm for 15min while being maintained at 4°C. The supernatant was collected and quantified using a BCA Protein Assay Kit (Thermo Fisher Scientific; Catalog # 23225). Protein extracts were also obtained from rat kidneys to serve as controls. The samples were mixed with LDS Sample Loading Buffer (Thermo Fisher Scientific; Catalog # 84788), heated for 10 min at 95°C, and loaded on mini-gels (15μg per well) (Thermo Fisher Scientific; NuPAGE® Novex® 10% Bis-Tris Protein Gels, Catalog # NP0315BOX). Electrophoresis was carried out at 120V for 2–2.5 h, and gels were electrotransferred to nitrocellulose membrane at a constant current of 0.1A for 2h. The blots were then blocked by 5% non-fat milk powder in Tris Buffered Saline (TBS) for 1 h at room temperature, and incubated overnight with primary antibodies to Slc26a1, Slc26a6, Slc26a7 and β-actin (internal control), separately (Table 1). After appropriate HRP-conjugated secondary antibodies were applied for 2 h at room temperature, the blots were washed and developed with substrate kits (Thermo Fisher Scientific; SuperSignal West Pico Chemiluminescent Substrate, Catalog # 34077; SuperSignal West Femto Maximum Sensitivity Substrate, Catalog # 34095). The immunoblotting experiment was conducted using the protein samples from each animal separately. Quantification of the relative intensity of the bands was conducted using NIH ImageJ software version 1.48. Mann-Whitney U test was used to detect the potential difference in protein-level gene expression between secretory- and maturation stage enamel formation (IBM SPSS Statistics 22.0, α = 0.05).

### Co-immunoprecipitation (Co-IP)

Maturation-stage enamel organ protein samples were obtained using the procedures described above, and were then lysed in 1xTBS with 1% Triton-100. After being pre-cleared by 20ul A/G agarose beads at 4°C for 1 h, the protein samples (50–100μg) were incubated with 1μg of primary antibody to Cftr (Table 1) at 4°C for 1 h, while being gently agitated. The samples were then mixed with 20μl A/G agarose beads and kept at 4°C overnight. The beads were washed extensively with Tris buffer (50mM Tris, 0.1% NP-40, pH 7.4), and the bound proteins were subject to Western blot analyses with the primary antibodies against Slc26a1, Slc26a6 and Slc26a7 (separately), and the appropriate secondary antibody (Table 1). Protocols for Western blot analysis have been described previously. Positive and negative controls were set up simultaneously with the experimental groups. The positive control was the pre-cleared total protein without subsequent immunoprecipitation procedures, while the negative control was prepared by skipping the step of applying the primary antibody against Cftr.

### Micro-CT analysis

Mandibles were isolated from 8-week-old *Slc26a1* ^*-/-*^ and *Slc26a7* ^*-/-*^ mice and their respective age-matched WT littermates. Four animals from each group were analyzed (n = 4 per group). The hemi-mandibles were air-dried for at least 7 days before micro-CT (μCT) analyses were conducted. The samples were scanned with a Siemens MicroCAT II at the Molecular Imaging Center of the University of Southern California. The acquisition settings were documented as previously described [30]. The reconstruction and the subsequent calculation of the relative density of fully mature enamel and dentin were performed with Amira 3D Visualization and Analysis Software 5.4.3 (FEI Visualization Science Group, Burlington, MA, USA). Two-tailed Student’s t tests were used to evaluate the potential statistical differences in the relative density and the thickness of enamel between *Slc26a1* ^*-/-*^ and their WT littermate controls, and *Slc26a7* ^*-/-*^ and their WT littermate controls, using IBM SPSS Statistics 22.0 (α = 0.05).

### Scanning electron microscopy (SEM), energy-dispersive X-ray spectroscopy (EDS) and hardness test

The hemi-mandibles used for SEM and microindentation analyses were extracted from 8-week-old *Slc26a1* ^*-/-*^ and *Slc26a7* ^*-/-*^ mice and their respective age-matched WT littermates (n = 6 per group). The samples were prepared and scanned as previously documented [23,30]. EDS was conducted on JEOL JSM-7001F to analyze the elemental composition of *Slc26a1* ^*-/-*^ and *Slc26a7* ^*-/-*^ enamel, with an accelerating voltage of 10 kV. The differences in atomic percent (At%) of elements Ca, P, O, C, Cl, Na and Mg between mutant (*Slc26a1* ^*-/-*^ and *Slc26a7* ^*-/-*^) and wild-type enamel were detected using two-tailed Student’s t tests in IBM SPSS Statistics 22.0 (n = 6 per group, α = 0.05).

## Results

### Up-regulation of Slc26a1, Slc26a6 and Slc26a7 during enamel maturation

**Fig 2:**
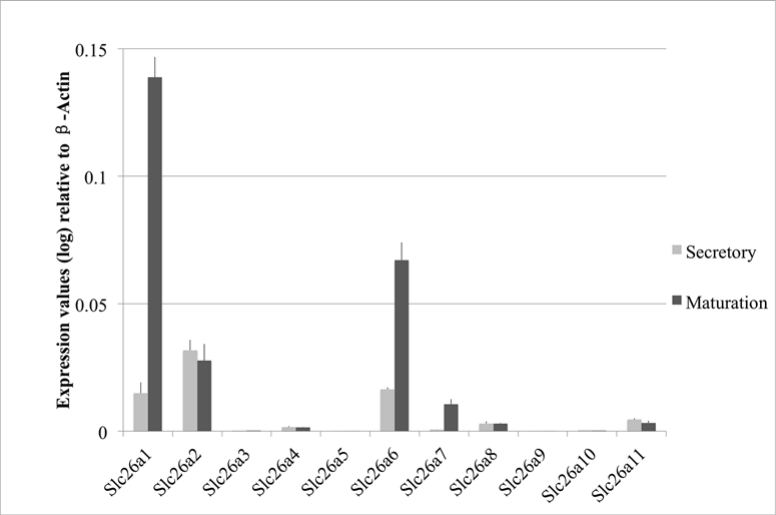
Real-time PCR analysis of Slc26a1, Slc26a6 and Slc26a7 expression during amelogenesis. The expression levels of Slc26a1, Slc26a6 and Slc26a7 were normalized to those of Beta-Actin and are presented in fold changes. The expression levels of Slc26a1, Slc26a6 and Slc26a7 were up-regulated by ~10.0, ~4.1 and ~15.3 fold, respectively, at maturation stage relative to secretory stage.

**Fig 3:**
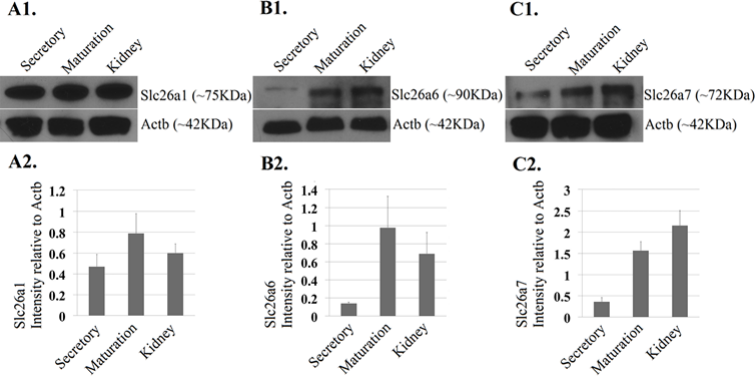
Western blot analysis of Slc26a1, Slc26a6 and Slc26a7. A1-C1. Protein-level expression of Slc26a1, Slc26a6 and Slc26a7 was detected by western blot analysis using samples obtained from both secretory- and maturation-stage enamel organs (4-week-old rat incisors). Protein samples extracted from kidney (4-week-old rat) were used as reference controls. The molecular weights for Slc26a1, Slc26a6 and Slc26a7 are 75kDa, 90kDa and 72kDa, respectively. Beta-Actin served as the control for sample loading. A2-C2. The intensities of the bands (relative to Beta-Actin) were measured using ImageJ. The average fold changes of Slc26a1, Slc26a6 and Slc26a7 at the protein level were ~1.6, ~6.1, ~4.2, respectively.

Based on our previous genome-wide miRNA and mRNA transcriptome analyses using RNA samples extracted from the secretory- and maturation-stage enamel organs of 4-week-old rat mandibular incisors, we showed that among all the Slc26 gene family members, Slc26a1, Slc26a6 and Slc26a7 are the only three that are differentially expressed between the two developmental stages (Table 2). In this current study we conducted real-time PCR analysis using the same RNA samples used previously for genome-wide mRNA transcriptome analysis [34]. We verified that Slc26a1, Slc26a6 and Slc26a7 were all significantly up-regulated at the mRNA level during maturation-stage tooth development (relative to secretory stage) (*P*<0.05; Fig 2), which is consistent with the results obtained from prior microarray analysis [34] (Table 2). The average fold changes for Slc26a1, Slc26a6 and Slc26a7 were ~ 15.4, ~ 3.9 and ~ 8.1, respectively (Fig 2). In order to assess Slc26a1, Slc26a6 and Slc26a7 protein expression levels, we performed Western blot analysis using protein samples obtained from secretory- and maturation-stage enamel organs of rat mandibular incisors, as well as from rat kidney (as a reference control tissue). At the protein level, Slc26a1, Slc26a6 and Slc26a7 all exhibited higher expression during maturation stage when compared to secretory stage (Fig 3, P<0.05). The average fold changes in the protein expression levels of Slc26a1, Slc26a6 and Slc26a7 were ~1.6, ~6.1, ~4.2, respectively (Fig 3), which were not as great as those calculated at mRNA levels with the exception of Slc26a6 (Fig 2), but there was a consistent directional change (mRNA and protein) for all three genes. In addition, Slc26a1 and Slc26a6 showed higher protein abundance in maturation-stage enamel organ than in kidney, while the trend was the opposite for Slc26a7 (Fig 3).

**Table 2:** Average fold changes of Slc26 gene family members during maturation stage relative to secretory stage based on genome-wide mRNA transcriptome analysis. (N/A, expression not detected; α = 0.05).

Gene symbol	Fold changes	*P* values
Slc26a1	39.8	0.000031
Slc26a2	-1.2	0.368
Slc26a3	1.2	0.391
Slc26a4	1.0	0.825
Slc26a5	1.1	0.711
Slc26a6	5.4	0.00016
Slc26a7	7.8	0.00013
Slc26a8	1.1	0.8
Slc26a9	-1.1	0.263
Slc26a10	N/A	N/A
Slc26a11	-1.5	0.278

### Localization of Slc26a1, Slc26a6 and Slc26a7 in the enamel organ

**Fig 4:**
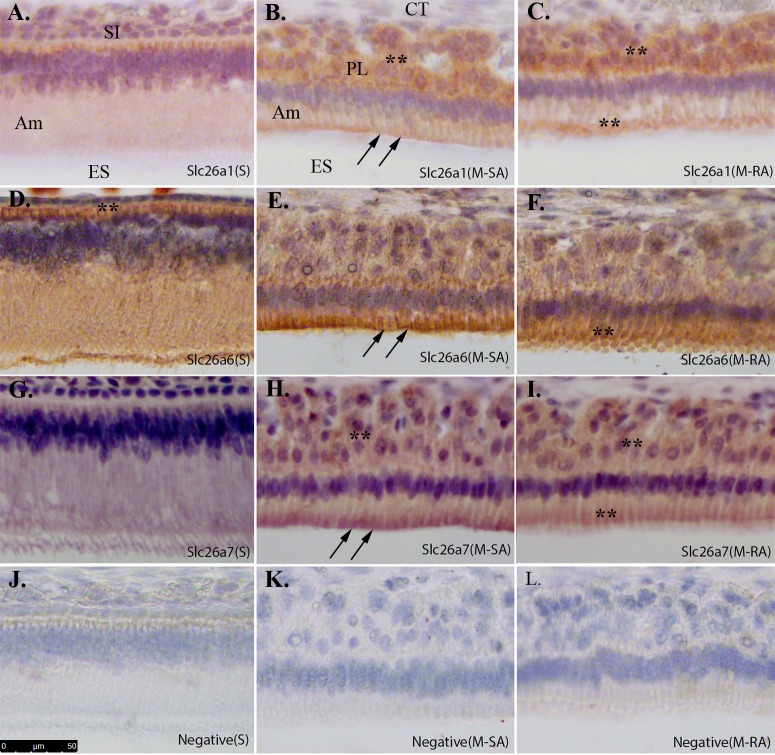
Immunoperoxidase immunostaining of Slc26a1, Slc26a6 and Slc26a7 in secretory- and maturation-stage enamel organ. Immunostaining procedures were applied to the sagittal sections prepared from paraffin-embedded 4-week-old rat mandibles. A. Slc26a1 in secretory-stage ameloblasts (S); B. Slc26a1 in smooth-ended ameloblasts at maturation stage (M-SA); C. Slc26a1 in ruffle-ended ameloblasts at maturation stage (M-RA); D. Slc26a6 in secretory-stage ameloblasts (S); E. Slc26a6 in smooth-ended ameloblasts at maturation stage (M-SA); F. Slc26a6 in ruffle-ended ameloblasts at maturation stage (M-RA); G. Slc26a7 in secretory-stage ameloblasts (S); H. Slc26a7 in smooth-ended ameloblasts at maturation stage (M-SA); I. Slc26a7 in ruffle-ended ameloblasts at maturation stage (M-RA); J-L. The sections that were incubated without antibodies served as negative controls for immunostaining. All images were collected under 20x magnification. Scale bar shown in Panel J (50μm). Slc26a1, Slc26a6 and Slc26a7 all showed expression on the apical membrane and/or within subapical cytoplasmic region (double black arrows). Positive staining in other regions was indicated by double black asterisks. SI—Stratum intermedium; Am—Ameloblast; ES—Enamel space; CT—Connective tissue; PL—Papillary layer.

Using sagittal sections prepared from 4-week-old rat mandibles, immunoperoxidase immunostaining was performed to clarify the expression patterns of Slc26a1, Slc26a6 and Slc26a7 in both secretory- and maturation-stage enamel organs. At secretory stage, Slc26a1 was mainly localized to the basal membrane of ameloblasts (Fig 4A). The localization of Slc26a6 within the secretory-stage enamel organ seemed to be more diverse than Slc26a1; it is expressed at both the basal membrane and apical membrane of ameloblasts (Fig 4D). The expression of Slc26a7 was barely detected by IHC in secretory-stage enamel organs (Fig G). In maturation-stage enamel organ cells Slc26a1, Slc26a6 and Slc26a7 were all expressed in both the ameloblasts and the papillary layer (Fig 4B, 4C, 4E, 4F, 4H and 4I), but the relative expression patterns varied. For Slc26a1, higher expression was observed in papillary layer than in ameloblasts (Fig 4B and 4C). In maturation-stage ameloblasts, Slc26a1 was localized to the apical and/or subapical domains of the cytoplasmic membrane (Fig 4B and 4C). The relative distribution of Slc26a6 in maturation-stage enamel organ cells differed from Slc26a1, with higher expression of Slc26a6 observed in the ameloblasts rather than in the papillary layer (Fig 4E and 4F). The localization of Slc26a6 in ameloblasts also varied between smooth-ended ameloblasts (SA) and ruffle-ended ameloblasts (RA), with a greater apical concentration of Slc26a6 seen in SA (Fig 4E compared to 4F). Slc26a7 is expressed both in the ameloblasts and papillary layer (Fig 4H and 4I) with a greater apical concentration seen in SA when compared to RA (Fig 4H compared to 4I).

**Fig 5:**
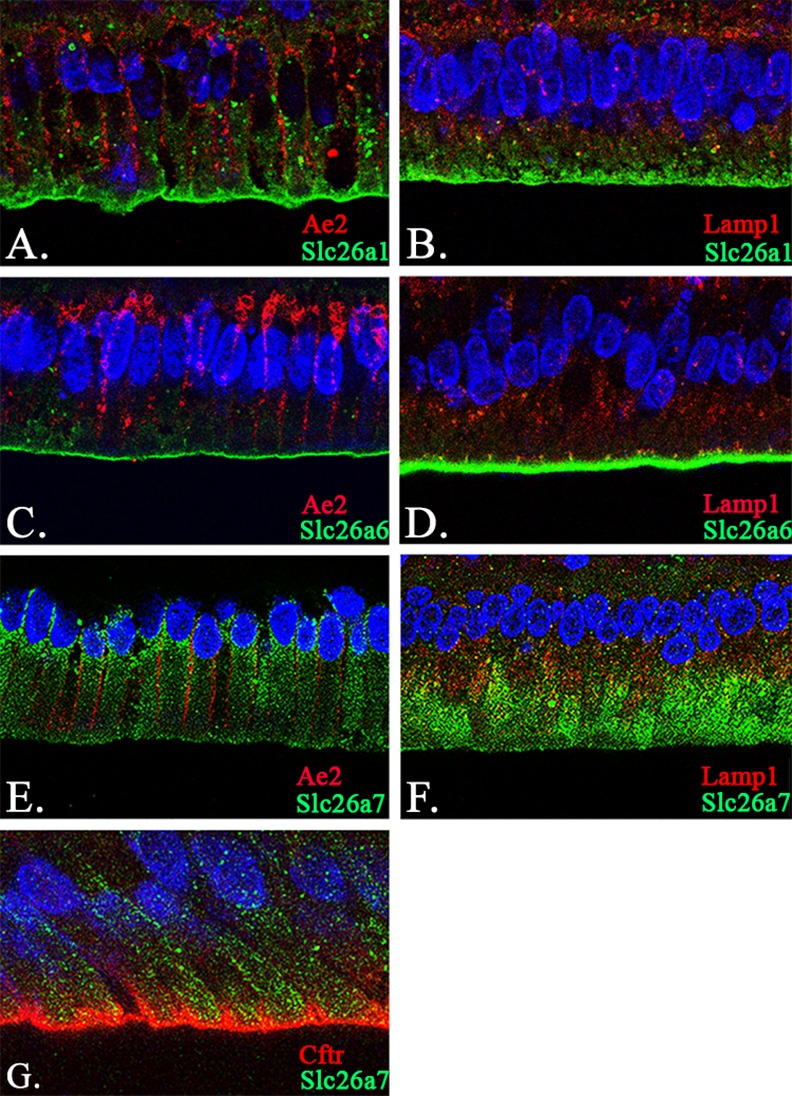
Co-localization analysis of Slc26a1, Slc26a6, Slc26a7 with Ae2, Lamp1 and Cftr. (A-B) Co-localization of Slc26a1 with Ae2 and Lamp1 in maturation-stage ameloblasts by confocal microscopy at 63x magnification. The signals of Slc26a1 were mainly seen on the apical membrane of maturation-stage ameloblasts (Panels A and B; Green). In contrast, Ae2 was localized to the basolateral membrane (Panel A; Red) and the basal pole (Panel C; Red), while Lamp1 showed a cytoplasmic and/or peri-nuclear distribution pattern in ameloblasts (Panel B; Red). The sections were stained with DAPI to highlight the nuclei (Panels A and B; Blue). (C-D) Co-localization of Slc26a6 with Ae2 and Lamp1 in maturation-stage ameloblasts. Slc26a6 exhibited a similar expression pattern to that of Slc26a1—on the apical membrane of maturation-stage ameloblasts (Panels C and D; Green). The fluorescence signals of Ae2 (Panel C; Red) and Lamp1 (Panel D; Red) were used as references. The sections were stained with DAPI to highlight the nuclei (Panels C and D; Blue). (E-G) Co-localization of Slc26a7 with Ae2, Lamp1 and Cftr in maturation-stage ameloblasts. The expression of Slc26a7 was found both on the apical membrane and within the cytoplasmic region (Panels E-G; Green). The fluorescence signals of Slc26a7 partially overlapped with those of Lamp1 (Panel F; Red) and Cftr (Panel G; Red), rather than Ae2 (Panel E; Red). The sections were stained with DAPI to highlight the nuclei (Panels E-G; Blue). The images were collected under confocal microscopy (63x magnification). Scale bar shown in Panel G (10μm).

With the purpose of acquiring deeper insight into the localization of Slc26a1, Slc26a6 and Slc26a7 in maturation-stage ameloblasts, we conducted dual immunofluorescence (IF) to establish the spatial localization of each of these three genes with respect to Slc4a2/Ae2, Lamp1 and Cftr. The expression patterns of Ae2, Lamp1 and Cftr during enamel maturation have been previously studied [2,8,13,14,18,26]. Based on the IF analysis, we showed that Slc26a1 and Slc26a6 exhibit similar distribution patterns during maturation stage, when they are both localized to the apical/subapical region of ameloblasts (Fig 5A–5D). Slc26a7 expression is localized to the apical/subapical membrane and the membrane of cytoplasmic vesicles of maturation-stage ameloblasts (Fig 5E, 5F and 5G). These data for Slc26a1, Slc26a6 and Slc26a7 are consistent with the observations seen in IHC (Fig 4). In ameloblasts the spatial localization of Ae2 is at the lateral membrane (Fig 5A, 5C and 5G), and Lamp1 is localized to the peri-nuclear region within the cytoplasm (Fig 5B, 5D and 5F). Cftr is localized to the apical membrane of maturation-stage ameloblasts (Fig 5G) [13]. Taken together, our data show that Cftr, Slc26a1 and Slc26a6 show significant colocalization patterns in maturation-stage ameloblasts.

### Slc26a1, Slc26a6 and Slc26a7 interact with Cftr

**Fig 6:**
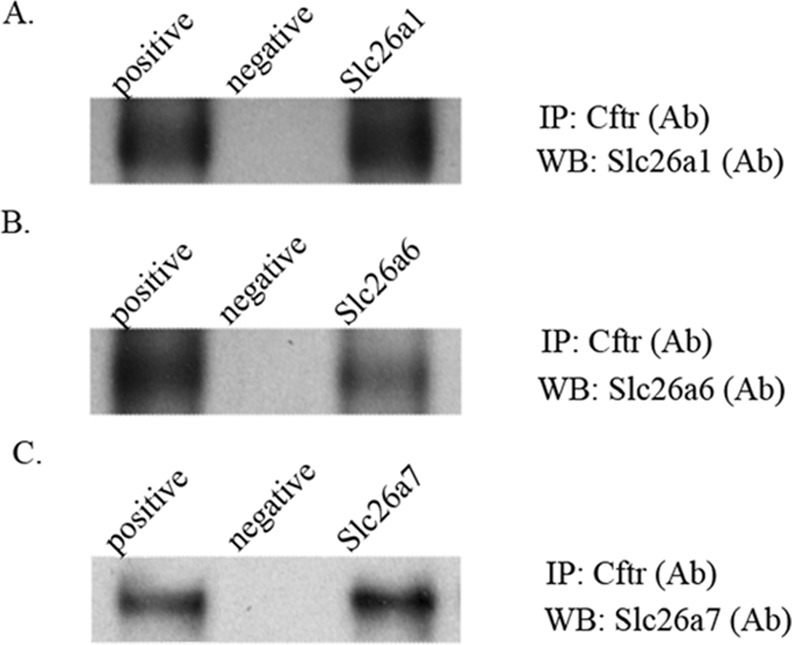
Co-immunoprecipitation (Co-IP) assay of Cftr with Slc26a1, Slc26a6 and Slc26a7. Co-IP was conducted using protein samples extracted from the maturation-stage enamel organs of 4-week-old rat incisors (50~100 μg initial input). The interaction complexes were pulled down by anti-Cftr antibody (Table 3). The subsequent western blot analyses were performed using primary antibodies to Slc26a1, Slc26a6 and Slc26a7 (Table 3). The positive control was the pre-cleared total protein without the following immunoprecipitation procedures, and the negative control was prepared by skipping the step of applying the antibody against Cftr.

As we have shown, Slc26a1, Slc26a6, Slc26a7 and Cftr are all localized to the apical membrane of maturation-stage ameloblasts (Figs 4 and 5). To investigate the hypothesis that the similarity in the expression patterns of Slc26a1, Slc26a6 and Slc26a7 to that of Cftr on the apical membrane of ameloblasts during enamel maturation may involve a direct physical interaction, we conducted co-immunoprecipitation (Co-IP) assays using protein samples obtained from maturation-stage enamel organs. We confirmed that from the protein complex pulled down by anti-Cftr antibody, Slc26a1, Slc26a6 and Slc26a7 are all able to be detected by the corresponding antibodies separately (Fig 6), indicating that there exist protein-protein interactions between Cftr and each of the three SLC26 genes studied. Positive and negative controls were included in the co-IP assays (Fig 6).

### Enamel phenotypes of Slc26a1 null and Slc26a7 null animals

**Fig 7:**
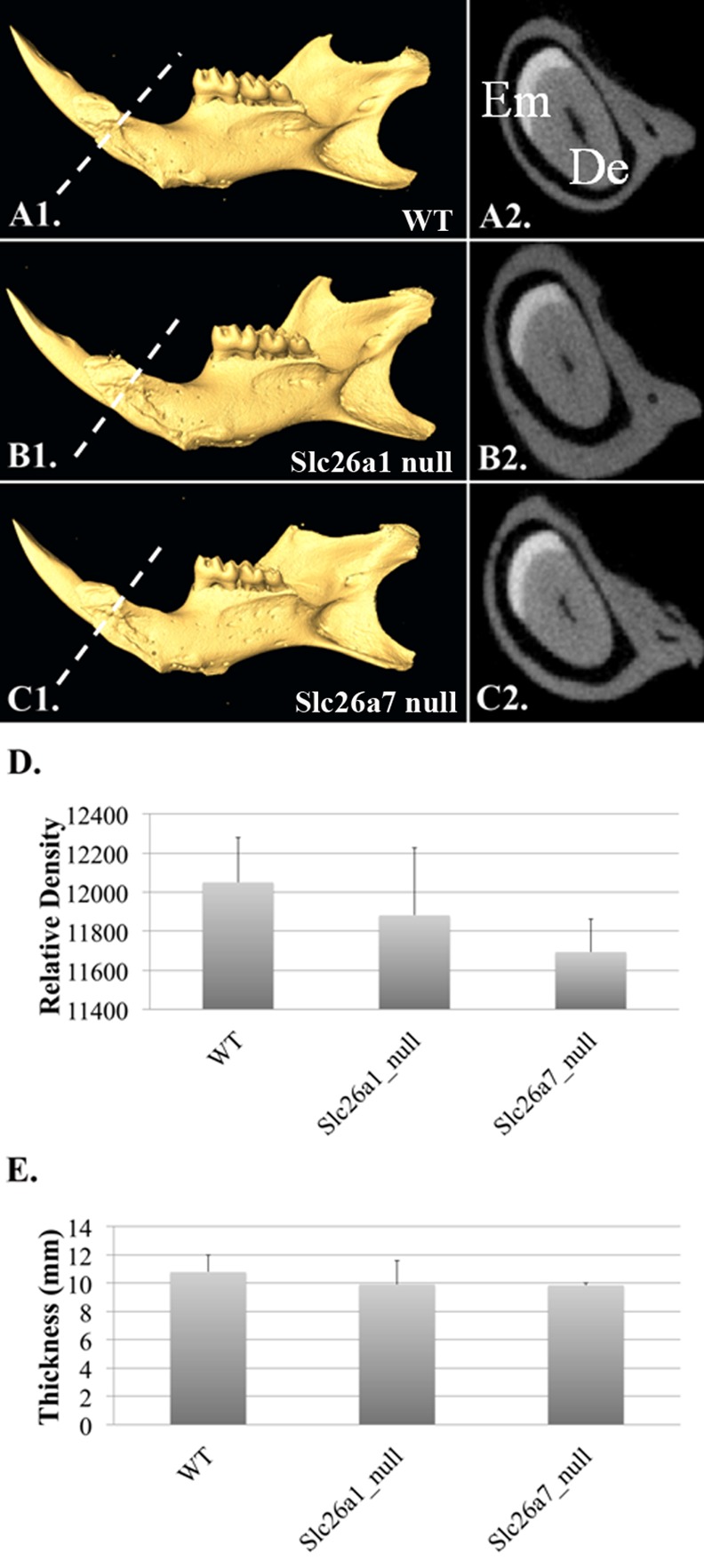
Micro-CT analysis of Slc26a1^-/-^ and Slc26a7^-/-^ mandibles. The mandibles from wild-type, *Slc26a1* null and *Slc26a7* null animals (at 8 weeks of age) were subject to micro-CT analysis (n = 3). The relative density and thickness of enamel on the labial incisor where the cortical bone enclosing just begins (A1-C2) were measured. There was no statistical difference between mutant and wild-type animals with respect to these two parameters (D-E). (Enamel Em, Dentin De)

**Fig 8:**
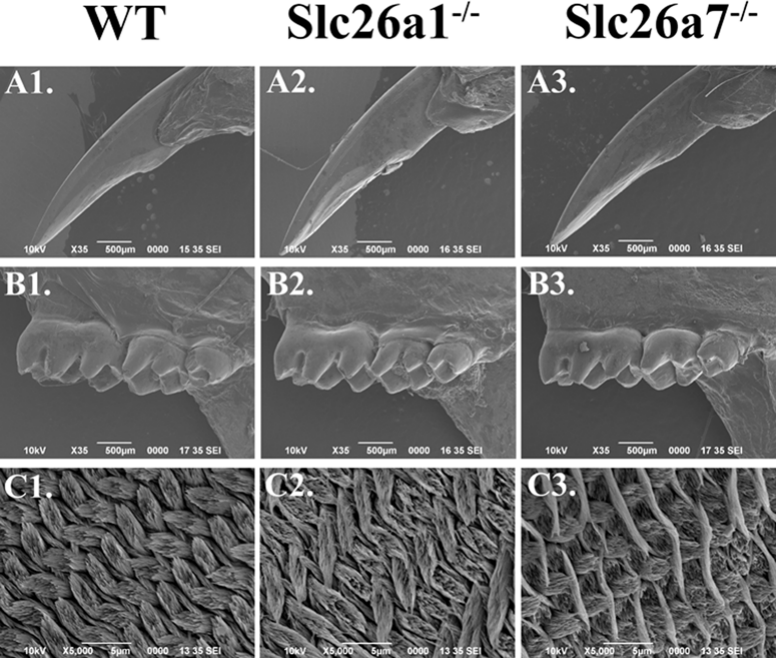
SEM images of mature enamel in Slc26a1^-/-^ and Slc26a7^-/-^ animals. The surface of the enamel of incisor and molars in *Slc26a1* ^*-/-*^ and *Slc26a7* ^*-/-*^ animals were similar to those of wild-type animals (A1-B3, magnification 35x). When the internal structures of enamel (incisor) were observed in coronal section (C1-C3, magnification 5000x), the enamel from mutant animals showed a mild disruption in rod density and diameter (C2-C3) compared with wild-type enamel (C1). The arrangement of the enamel rod and inter-rod structures (incisor) did not seem to be impacted significantly by the deletion of *Slc26a1* or *Slc26a7* (C1-C3).

**Fig 9:**
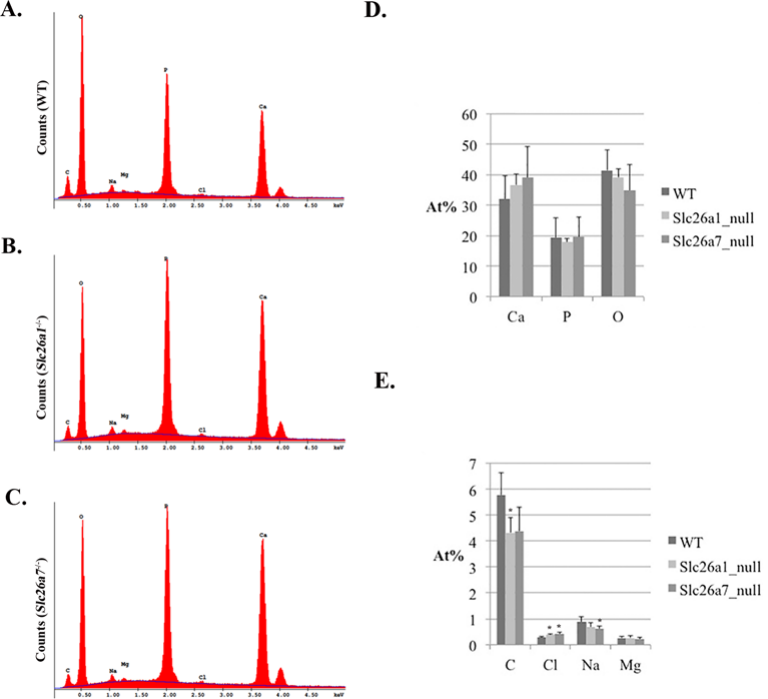
EDS analysis of mature enamel Slc26a1^-/-^ and Slc26a7^-/-^ animals. (A-C) EDS spectrum of mature enamel in wild-type, *Slc26a1* ^*-/-*^ and *Slc26a7* ^*-/-*^ animals (n = 6 per group). (D) No statistically significant differences were detected in the At% of Ca, P and O between mutant and wild-type enamel. (E) The At% of Cl increased significantly by ~34% in both *Slc26a1* ^-/-^ (*P* = 0.012) and *Slc26a7* ^-/-^ (*P* = 0.035) enamel. There was a significant decrease in the At% of C in *Slc26a1* ^-/-^ enamel (*P* = 0.028). A similar difference in the At% of C was detected between *Slc26a7* ^-/-^ and wild-type enamel, but was only marginally significant (*P* = 0.078). The At% of Na in *Slc26a7* ^-/-^ enamel also significantly decreased (*P* = 0.028).

All teeth prepared for micro-CT and SEM scanning were dissected from 8-week-old animals (*Slc26a1-/-*, *Slc26a7-/-* and their wild-type littermates). Generally, there were no remarkable differences between the mutant (*Slc26a1-/-* or *Slc26a7-/-*) and the wild-type teeth in terms of gross anatomy, microstructure or hardness of enamel (Figs 7 and 8, Table 3). We used Amira 3D Visualization and Analysis Software 5.4.3 to delineate enamel in scanned teeth and calculate its relative density. Moreover, we measured the thickness of enamel of incisors from 3D-reconstructed teeth at the point where the enclosing cortical bone of incisors terminates. These parameters obtained from *Slc26a1* ^*-/-*^ and *Slc26a7* ^*-/-*^ animals were compared separately with those from wild-types. However, no statistically significant differences were detected (P>0.05) (Fig 7). Nevertheless, the elemental composition of mutant enamel showed significant changes when compared with wild-type enamel. The Atomic percentages (At%) of Cl increased by ~34% in both *Slc26a1* ^*-/-*^ (P = 0.012) and *Slc26a7* ^*-/-*^ (P = 0.035) enamel (Fig 9A–9C and 9E). There was a decrease of ~24% in the At% of C in *Slc26a1* ^*-/-*^ enamel (P = 0.028), and the difference in the At% of C between *Slc26a7-/-* and wild-type enamel was marginally significant (P = 0.078) (Fig 9E). In addition, the At% of Na in *Slc26a7* ^*-/-*^ enamel also decreased significantly (P = 0.028) (Fig 9E).

**Table 3:** Enamel and dentin Vickers microhardness of mutant animals.

	Enamel, mean (SD)	Dentin, mean (SD)
WT (control for *Slc26a1* null)	2.3 (0.3)	0.66 (0.02)
*Slc26a1* null	2.0 (0.3)	0.62 (0.02)
WT (control for *Slc26a7* null)	2.2 (0.1)	0.63 (0.02)
*Slc26a7* null	2.5 (0.1)	0.58 (0.02)

The means and standard deviations (SD) of enamel and dentin microhardness are listed in units of GPa for mutant animals and their wild-type littermates. The sample size for each experimental group was 6, with 10 repetitions used to describe each tooth, and the individual tooth means were averaged. Differences in hardness between wild type and null animals were small, if present. It appears that biomechanical function, as measured by hardness, was unaffected by the knockout of a single transporter. Likewise, light microscopy revealed no macro- or micro-level differences in form, structure or organization between wild-type and null animals.

### Compensatory gene expression in Slc26a1 null and Slc26a7 null animals

**Fig 10:**
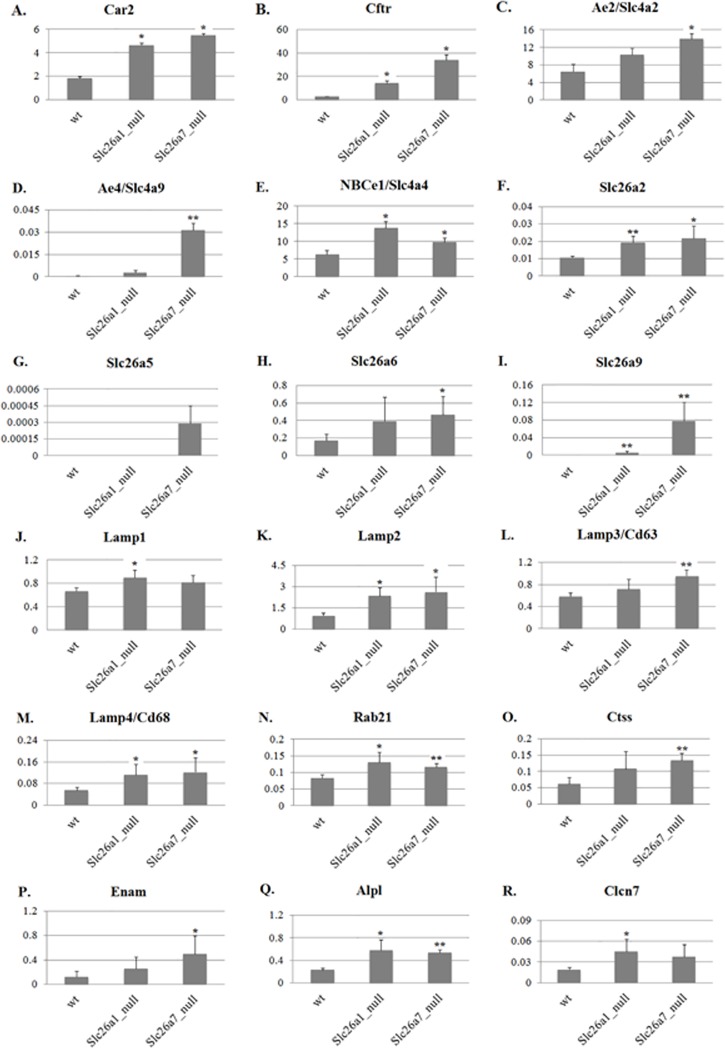
Up-regulated genes in Slc26a1^-/-^ and Slc26a7^-/-^ animals compared with wild types. Most genes that showed significant changes in expression were up-regulated (Panels A-S), indicating a compensatory effect induced by the deletion of Slc26a1 or Slc26a7. The expression values of differentially expressed genes were normalized to those of *Beta-Actin*. For all the two-tailed t tests used, the significance level was 0.05. * <0.05; ** <0.01.

**Fig 11:**
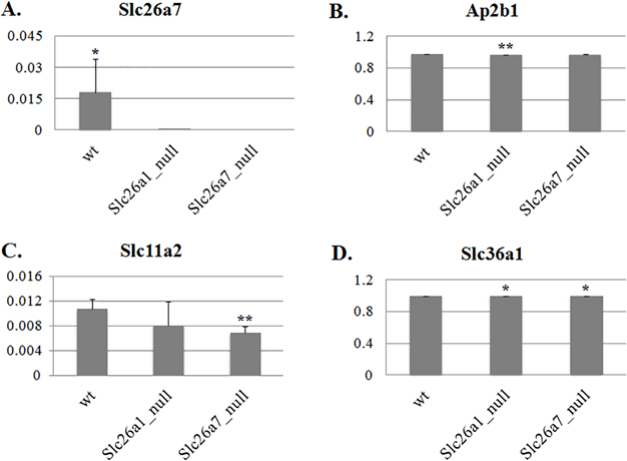
Downregulated genes in Slc26a1^-/-^ and Slc26a7^-/-^ animals compared with wild types. The expression values of differentially expressed genes were normalized to those of *Beta-Actin*. For all the two-tailed t tests used, the significance level was 0.05. * <0.05; ** <0.01.

Since we observed no overt abnormalities in mature enamel from either *Slc26a1* ^*-/-*^ or *Slc26a7* ^*-/-*^ animals, we sought to investigate the changes in the expression profiles of genes that have a similar biological function to the anion exchangers Slc26a1 and Slc26a7 during maturation-stage enamel formation in mutant animals (complete list of interrogated genes provided in S2 Table). Total RNA samples used for real-time PCR analysis were extracted from the maturation-stage enamel organs of 4-week-old mutants (*Slc26a1* ^*-/-*^ and *Slc26a7* ^*-/-*^) and their age-matched WT littermates. Among the 41 genes examined, 22 genes showed differential expression (18 up-regulated and 4 down-regulated) in *Slc26a1* ^*-/-*^ and/or *Slc26a7* ^*-/-*^ animals compared with WT (*P*<0.05; S2 Table, Figs 10 and 11). Many of the up-regulated genes have been well characterized as being involved in either pH regulation (i.e., *Cftr*, *Car2*, *Ae2* and *NBCe1*) or endocytosis (i.e., the lysosomal-associated membrane proteins Lamp1, Lamp2, Cd63 and Cd68, and others such as Clcn7, Rab21 and Ctss) during enamel maturation (Table 3, Fig 10) [7,8,12–32,43–53]. It is noteworthy that *Cftr*, *Slc4a9/Ae4*, *Slc26a5* and *Slc26a9* exhibited the largest scales of fold changes (>5) among the up-regulated genes in both mutant animal groups (S2 Table, Fig 10). These data provide strong evidence that enamel organ cells initiate a compensatory up-regulation of anion exchanger gene expression following the deletion of either Slc26a1 or Slc26a7. Finally, the expression of some of the SLC4 and Slc26 gene family members was not detected in the enamel organs of mutant mice strain, namely *Slc4a5/NBCe2*, *Slc26a3*, *Slc26a8* and *Slc26a10* (S2 Table).

## Discussion

During maturation-stage of enamel development, ameloblasts function to remove organic protein debris from extracellular enamel matrix [7,54] and to deposit inorganic ions into the enamel area [3], so that the crystal structures in enamel achieve their final width and thickness, rendering the dental enamel fully mature and functional. In the extracellular matrix, the mineral deposition, crystal growth and protease activities are presumed to be highly pH-dependent [3,55], while in the lysosomal lumen, an acidic pH is necessary for the activation of hydrolytic enzymes and the degradation of internalized macromolecules [7,9,56]. Thus, these two interconnected key processes at maturation stage require the tight control of pH to maintain either luminal and extracellular acid-base balance, which is mediated by the diffusion of ions across various biological membranes [2,18]. Although the detailed mechanisms of maturation-stage pH regulation are yet to be elucidated, previous investigations have suggested the essential role of several genes, such as *CFTR*, *AE2*, *NBCe1*, *CA2*, *CA6*, *NHE1* and *CLCN7*, in enamel maturation [2,7,12–15,18,20,21,23,25,26,29–32,43,45,46,57]. In this study, we provided experimental evidence to show that *SLC26A1/SAT1*, *SLC26A6/PAT1* and *SLC26A7/SUT2* are novel candidate genes that are involved in the pH regulation processes during maturation-stage tooth development.

**Fig 12:**
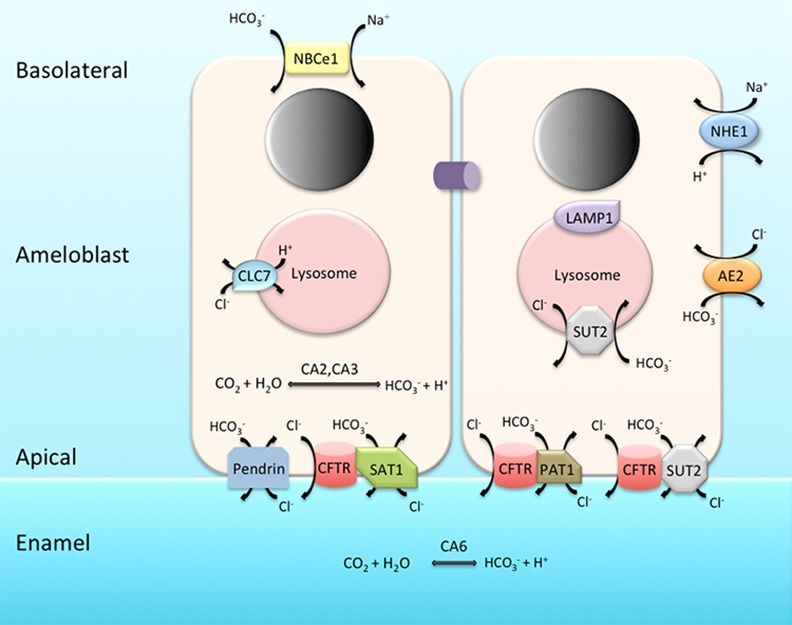
Schematic diagram depicting the distribution of major pH regulators in maturation-stage ameloblasts. Cftr, Slc26a1, Slc26a4, Slc26a6 and Slc26a7 are localized to the apical membrane and Slc26a1, Slc26a6 and Slc26a7 physically interact with Cftr to form regulation complexes. Slc26a7 is also found on the endo-lysosomal membrane. Ae2, Nhe1 and NBCe1 are localized basolaterally. Clcn7 is expressed on the endo-lysosomal membrane. CA2 exhibits intracellular distribution whereas CA6 functions in extracellular enamel matrix. Lamp1 in this image was used as a marker of late lysosomes.

The cellular localization patterns of genes that participate in the pH regulation processes during enamel maturation have been shown to be quite diverse, which may be direct reflections of their functional roles. For example, Cftr serves as a chloride channel mainly on the apical plasma membrane of the maturation-stage ameloblasts, and thus is considered a regulator of pH during rapid crystal growth [13] (Figs 6 and 12); NBCe1 and Ae2 localize to the basolateral membrane of ameloblasts at maturation stage, and the bicarbonate transport activity mediated by NBCe1 and Ae2 is thought to be critical to intracellular pH homeostasis [14,20,26] (Fig 12); Clcn7 is identified as being localized to the intracellular organelles, presumably at the late endosome/lysosomal membrane, suggesting that Clcn7 might help to accumulate protons within the lumen of these organelles and forms part of the endocytotic apparatus in maturation-stage ameloblasts [7] (Fig 12). According to our results from immunoperoxidase immunostaining and immunofluorescence, the distributions of Slc26a1, Slc26a6 and Slc26a7 are similar—they all localize to the apical membrane of maturation-stage ameloblasts, and in the case of Slc26a7, to the subapical and cytoplasmic region (Figs 5 and 6). These data indicate that the anion exchanger activities of Slc26a1, Slc26a6 and Slc26a7 on the apical membrane of maturation-stage ameloblasts may respond to the intracellular and/or extracellular pH changes, and regulate pH values by secreting bicarbonate into enamel matrix to neutralize protons (Fig 12). In addition to the apical membrane, Slc26a7 is also seen in the cytoplasmic region within maturation-stage ameloblasts (Fig 6E–6G). It is highly possible that the intracellular localization of Slc26a7 is to the membranous structures in the early and/or late endo-lysosomal pathway (Fig 12). This is mainly evidenced by the observations that there are partial overlaps in the fluorescence signals of Lamp1 (late endo-lysosome-localized [8,58]) and Slc26a7 in co-localization assays, and that the overlaps reside in the subapical and middle regions instead of the peri-nuclear region of the cytosol (Fig 6F). In other functional cell types, the localization of Slc26a7 also demonstrates diversity as observed in maturation-stage ameloblasts. For example, in the kidney, Slc26a7 co-localizes with AE1 at the basolateral or subapical membrane, and with Tfrc at the endosomal membrane of A-intercalated cells in the renal outer medullary collecting duct (OMCD) [35,59]. The distinct distribution pattern of Slc26a7 in maturation-stage ameloblasts lends support to its potential role in regulating pH within the lysosomal lumen, which might be similar to that of Clcn7, in addition to the pH regulation presumed to be functioning in extracellular enamel matrix.

The phenomenon that several ion transporters/channels with similar physiological functions and cellular localizations interact with one another to form united protein complexes has been reported in multiple areas of biomedical research. In most cases when Cftr interacts with Slc26s, Cftr seems to serve as a hub for these potential interaction complexes [60–66]. One example is that Slc26a3, Slc26a6 and Slc9a3r1 co-localize with Cftr in the midpiece of mouse sperm, and the protein complex formed by Cftr with Slc26a3, Slc26a6 and Slc9a3r1 functions primarily to mediate transmembrane transport of chloride, which is critical for sperm capacitation [61]. In cochlear outer hair cells (OHCs), the physical interaction between Cftr and Slc26a5, which is localized to the lateral membrane of OHCs, has potential electrophysiological significance [64]. Additionally, in human bronchial cell lines, functional CFTR contributes to the functions of SLC26A9 as an anion conductor [60]. Based on our data from Co-IP, we presented evidence that physical interactions exist between Cftr and Slc26 gene family members Slc26a1, Slc26a6 and Slc26a7 in maturation-stage ameloblasts, and the functional complexes may be localized to the apical membrane where the expression of these genes is identified (Figs 5 and 6 and 12). At this stage, we did not seek to investigate the potential interactions between Cftr and other important pH regulators, such as Ae2, NBCe1 and Clcn7, mainly because of their distinct locations within the milieu of the ameloblast (Fig 12). However, it is unreasonable to rule out the possibility that physical interactions involving Cftr and other related genes do exist, as these pH regulators may exhibit mobility during ameloblast modulation cycles [21]. Therefore, it could be speculated that Cftr might interact with a broader range of pH regulators, and that the pH regulation process during enamel maturation might be achieved by the coordination of functional protein complexes that are far more sophisticated than expected.

Mutations in the pH regulators that have been identified to be functional during enamel maturation, such as Cftr, Ae2 and NBCe1, often result in severe AI-like enamel/tooth phenotypes [2,12–15,18,20,23,26,31,32]. In our study, deletion of Slc26a1 and Slc26a7 led to an increase of Cl and a decrease of C in the elemental composition of enamel matrix (Fig 9E), which is consistent with our hypothesis about the functional roles of Slc26a1 and Slc26a7 as anion exchangers of Cl^-^ (intracellular-oriented) and HCO_3_^-^ (extracellular-oriented) (Fig 12). However, no significant abnormalities in mature enamel structures or hardness were observed in Slc26a1 or Slc26a7 mutant animals (Figs 7 and 8, Table 2), although impaired physiological functions were documented in other organs [39,42], suggesting that there might be functional redundancy of Slc26a1 and Slc26a7 during amelogenesis. To investigate this hypothesis, we conducted real-time PCR reactions to detect potential changes in gene expression in mutant animals. While a small number of genes were selected for qPCR analysis, the selection was made based on previous studies showing that these genes or other members within the same gene family are involved in ion transport, pH regulation and endocytotic pathways during maturation-stage amelogenesis [2,7,12–18,20–24,26,27,29,31,32,45,47,55]. The results showed that deletion of Slc26a1 or Slc26a7 induced upregulation of multiple genes with similar functional roles in maturation-stage pH regulation and endocytotic pathway (Fig 10, S2 Table). The up-regulated genes can be roughly subdivided into three categories: 1) those that have been relatively well characterized, such as *Car2*, *Cftr*, *Ae2*, *NBCe1*, *Slc26a6*, *Clcn7*, *Lamp1-Lamp4*, *Rab21* and *Ctss* [2,7,12–18,20–24,26,27,29,31,32,45,47,55]; 2) those that are not expressed/differentially expressed in normal enamel maturation, such as *Slc26a2*, *Slc26a5*, *Slc26a9* and *Ae4* [43]; 3) other related genes, such as *Alpl* and *Enam*. Based on the features of these up-regulated genes, it is reasonable to conclude that there were strong compensatory reactions in response to the deletion of Slc26a1 or Slc26a7 in mutant animals. Absence of enamel phenotypes is hardly a novel discovery when the genes involved in maturation-stage pH regulation and endocytotic pathway are deleted, and can often be explained by the compensatory effect from other genes [7,67,68]. A final point to be noted is that compared with wild-type animals, the expression of Slc26a7 was downregulated by ~5 fold in Slc26a1 null animals (Fig 11, S2 Table). Previous investigations on *Car2* null animals demonstrated that Car2 deficiency decreases the expression of Slc26a4, Slc26a7 and Ae1 at the mRNA level in kidney collecting ducts [69]. The authors proposed that changes in the expression patterns of Slc26a4, Slc26a7 and Ae1 might be attributed to increased level of apoptosis, which could result from disturbance of pH homeostasis induced by Car2 deletion [69]. Similar explanations may or may not apply in case of decreased *Slc26a7* transcripts in *Slc26a1* null animals, and the interplay between Slc26a1 and Slc26a7 during enamel maturation warrants further investigation.

In summary, Slc26a1, Slc26a6 and Slc26a7 participate in maintaining the acid-base balance during amelogenesis, although deletion of Slc26a1 or Slc26a7 fails to induce abnormalities in enamel phenotypes. Moreover, Slc26a1, Slc26a6 and Slc26a7 contribute to the formation of more sophisticated functional complexes involving other stage-specific pH regulatory proteins, which may shed light on future investigations into the pathophysiological mechanisms of enamel development and health.

## Supporting Information

S1 Table Rat and mouse specific primers for qPCR and cDNA analyses. (XLSX)

S2 Table qPCR analysis of gene expression changes in Slc26a1 null and Slc26a7 null animals. (XLSX)
